# Dental pulp pluripotent-like stem cells (DPPSC), a new stem cell population with chromosomal stability and osteogenic capacity for biomaterials evaluation

**DOI:** 10.1186/s12860-017-0137-9

**Published:** 2017-04-21

**Authors:** Raquel Núñez-Toldrà, Ester Martínez-Sarrà, Carlos Gil-Recio, Miguel Ángel Carrasco, Ashraf Al Madhoun, Sheyla Montori, Maher Atari

**Affiliations:** 1 Regenerative Medicine Research Institute, Universitat Internacional de Catalunya, Barcelona, Spain; 2 Chair of Regenerative Implantology MIS-UIC, Barcelona, Spain; 3 Area of Pathology, Universitat Internacional de Catalunya, Barcelona, Spain; 4 Research Division, Dasman Diabetes Institute, Dasman, Kuwait; 5 Surgery and Oral Implantology Department, Universitat Internacional de Catalunya, Barcelona, Spain

**Keywords:** Dental pulp, Stem cells, Pluripotency, Genetic stability, Osteogenic differentiation, Biomaterials

## Abstract

**Background:**

Biomaterials are widely used to regenerate or substitute bone tissue. In order to evaluate their potential use for clinical applications, these need to be tested and evaluated in vitro with cell culture models. Frequently, immortalized osteoblastic cell lines are used in these studies. However, their uncontrolled proliferation rate, phenotypic changes or aberrations in mitotic processes limits their use in long-term investigations. Recently, we described a new pluripotent-like subpopulation of dental pulp stem cells derived from the third molars (DPPSC) that shows genetic stability and shares some pluripotent characteristics with embryonic stem cells. In this study we aim to describe the use of DPPSC to test biomaterials, since we believe that the biomaterial cues will be more critical in order to enhance the differentiation of pluripotent stem cells.

**Methods:**

The capacity of DPPSC to differentiate into osteogenic lineage was compared with human sarcoma osteogenic cell line (SAOS-2). Collagen and titanium were used to assess the cell behavior in commonly used biomaterials. The analyses were performed by flow cytometry, alkaline phosphatase and mineralization stains, RT-PCR, immunohistochemistry, scanning electron microscopy, Western blot and enzymatic activity. Moreover, the genetic stability was evaluated and compared before and after differentiation by short-comparative genomic hybridization (sCGH).

**Results:**

DPPSC showed excellent differentiation into osteogenic lineages expressing bone-related markers similar to SAOS-2. When cells were cultured on biomaterials, DPPSC showed higher initial adhesion levels. Nevertheless, their osteogenic differentiation showed similar trend among both cell types. Interestingly, only DPPSC maintained a normal chromosomal dosage before and after differentiation on 2D monolayer and on biomaterials.

**Conclusions:**

Taken together, these results promote the use of DPPSC as a new pluripotent-like cell model to evaluate the biocompatibility and the differentiation capacity of biomaterials used in bone regeneration.

**Electronic supplementary material:**

The online version of this article (doi:10.1186/s12860-017-0137-9) contains supplementary material, which is available to authorized users.

## Background

The increase in life expectancy has been associated with a rise in the number of bone-grafting procedures for diseases such as osteoporosis, arthritis, tumors and trauma; placing an even larger demand on the healthcare system to replace and restore bone loss [1]. Recently, a great deal of efforts has focused in the field of bone tissue engineering, and, particularly, in the area of stem cell biology and how to modulate their behavior through environmental cues [1].

Biomaterials have been shown to allow the guidance of stem cells in vitro as well as in vivo. In order to assess their biocompatibility as well as their ability to differentiate cells into specific lineages, these need to be tested in an in vitro cell culture model. For this purpose, many established cell lines and models have emerged to address the surge in research in this field [2]. For instance, for bone related biomaterials, most studies have examined their osteogenic potential using immature osteoblasts, immortalized cell lines or mesenchymal stem cells among others. Primary cells, such as lineage-specific osteoblasts, can be isolated and cultivated relatively easily; however, they have a limited lifespan [3]. Immortalized cell lines, such as the human sarcoma osteogenic cell line (SAOS-2) have been frequently used in applied biology since they are from human origin while providing unlimited number of cells [2, 4]. Nevertheless, these cell lines, due to their cancer origin, usually possess phenotype changes between passages, aberrations in mitotic processes and lack of growth inhibition, which limits their use in long-term investigations [5]. On the other hand, mesenchymal stem cells (MSC), which can be isolated from many adult tissues, are an attractive cell source for tissue engineering. These cells are self-renewable with a high proliferative capacity and possess a multi-lineage differentiation potential [6]. However, long-term MSC culture conditions, for their maintenance and expansion, cause morphological and immune-phenotypical changes which lead to cell senescence and alternations in their differentiation potential. For example, morphological abnormalities, cellular enlargement, miss expression of specific surface markers and an ultimate growth inhibition are associated with MSC that are cultured beyond passage 12 [7]. Therefore, there is a need to find a cell type with genetic stability and stemness characteristics to be used to evaluate biomaterials in cell therapy applications.

The dental pulp is an accessible niche housing neural crest-derived stem cells. This niche contains several populations of dental pulp stem cells (DPSC), with different properties. The first characterized population was the dental pulp mesenchymal stem cells (DPMSC), with multi-potential capability. Recently, a new stem cell population from the human dental pulp of third molars has been isolated. These cells, named dental pulp pluripotent-like stem cells (DPPSC) have particular culture conditions and, unlike DPMSC, express pluripotency markers until late passages and are able to differentiate into cells from the three germ layers (endoderm, mesoderm and ectoderm) [8, 9]. We therefore consider that DPPSC could be a promising cell population that can be used to evaluate the biological properties of biomaterials. The use of differentiated cells, e.g. osteoblast cells, limits the relevance of the biomaterial since it is already expected that the biomaterial will allow expression of osteogenic markers. For this purpose, the use of pluripotent cells that can potentially differentiate into any lineage can be properly guided by the biomaterial and hence demonstrating the efficiency of the biomaterial [10]. While this is the main purpose of pluripotent stem cells, up to date, embryonic stem cells and induced pluripotent stem cells (iPSC) have limited applications for biomaterials testing due to ethical reasons or low efficient transfections [10]. Hence, DPPSC might be used in order to overcome the current limitations of specific cell lineages or other pluripotent stem cells.

One of the key objectives of bone tissue engineering is the enhancement of stem cell mediated osteogenic differentiation under three-dimensional (3D) scaffold conditions to mimic engineering of clinically applicable bone constructs [1, 11]. In this way, scaffolds provide suitable support for cellular infiltration, migration, as well as, proper cell proliferation and differentiation [12]. Scaffolds are manufactured from several biomaterials including metals, ceramics, synthetic polymers or natural polymers. Currently, the components of the extracellular matrix play an important role as natural substrates for in vitro cells in cultures [13]. In this sense, collagen is regarded as an ideal scaffold for tissue engineering, as it provides support to connective tissues [14–16]. Collagen type I based materials are extensively used for basic cell culture applications, as well as in the fields of bioreactor technology and tissue engineering [17–19]. On the other hand, titanium and titanium alloys are primarily used in bone implant materials. Titanium has been widely used in medical practice, showing excellent biocompatibility and safety [20]. Hence, tissue compatibility, osseointegration and functional maintenance of functions are fundamental criteria for the long-term success of endosseous dental implants [21].

Therefore, the main purpose of this study was to assess the biocompatibility and the osteogenic capacity of DPPSC in the presence of different types of biomaterials used in bone regeneration studies, such as metals or natural scaffolds.

## Methods

### Patient selection and ethics statement

The third molars of healthy patients were extracted for orthodontic reasons and 6 different patients of different sexes and ages (14-21 years old) were selected.

The procedure and all experiments of this study were performed in accordance with the guidelines on human stem cell research issued which was approved by the Committee on Bioethics of the Universitat Internacional de Catalunya, with the study code: BIO-ELB-2013-03.

### Isolation of DPPSC and DPMSC from third molars

In this study, DPPSC and DPMSC were isolated from the same dental pulps as previously described [22]. Briefly, the molars were cleaned using gauze soaked in 70% ethanol previously the extraction of the dental pulp. Then, the dental pulp tissues were disaggregated by digestion with collagenase type I (3 mg/ml; Sigma) for 60 min at 37 °C. After washing twice with DPBS (Sigma), isolated cells were cultured in two different mediums and densities in order to separate DPPSC from DPMSC. DPPSC and DPMSC were maintained and expanded under different culture conditions until passage 15.

### Culture of DPPSC

The culture medium for DPPSC consisted of 60% DMEM-low glucose (Life Technologies) and 40% MCDB-201 (Sigma) supplemented with 1X SITE (Sigma), 1X LA-BSA (Sigma), 10^-4^M ascorbic acid 2-phosphate (Sigma), 1% penicillin/streptomycin (Life Technologies), 2% FBS (Biochrom), 10 ng/ml hPDGF-BB (R&D Systems) and 10 ng/ml EGF (R&D Systems). Flasks were pre-coated with 100 ng/ml fibronectin for one hour at 37 °C in 5% CO_2_ incubator. During the 2 weeks of primary culture, the medium was changed every 4 days. Cells were passaged when they were at 30% confluence by adding 0.25% trypsin-EDTA (Life Technologies) and then they were cultured at a density of 100 cells/cm^2^.

### Culture of DPMSC

The culture medium for DPMSC consisted of DMEM-high glucose (Life Technologies), 10% FBS (Biochrom) and 1% penicillin/streptomycin (Life Technologies). The medium was changed every 4 days during the first 2 weeks of primary culture. To propagate DPMSC, cells were detached at 80% confluence by the addition of 0.25% trypsin-EDTA (Life Technologies) and reseeded at a density of 2x10^3^ cells/cm^2^.

### Culture of SAOS-2

The commercially available SAOS-2 cells at passage 10 (Sigma) were seeded at density of 10^3^cells/cm^2^ in DMEM-high glucose (Life Technologies) supplemented with 10% FBS (Biochrom), 2 mM L-glutamine and 1% penicillin/streptomycin (Life Technologies), at 37 °C in a 5% CO_2_ incubator. The medium was changed every 3 days. After reaching 90% confluence, cells were detached by the addition of 0.25% trypsin-EDTA (Life Technologies).

### Osteogenic differentiation on 2D

DPPSC isolated from three different patients at passages 1, 5 and 10 were osteogenetically stimulated for 21 days. The osteogenic medium contained α − MEM (Gibco) containing 10% heat-inactivated FBS (Biochrom), 10 mM β-glycerol phosphate (Sigma), 50 μM L-ascorbic acid (Sigma), 0.01 μM Dexamethasone (Sigma) and 1% penicillin/streptomycin (Life Technologies) solution. Cells were cultured on 24 well culture plates at a cell density of 5 × 10^3^ cells/cm^2^. The cell line SAOS-2 were used as a control and seeded at the same density. The medium was changed every 2 days.

### Osteogenic differentiation on biomaterials

**Fig. 1:**
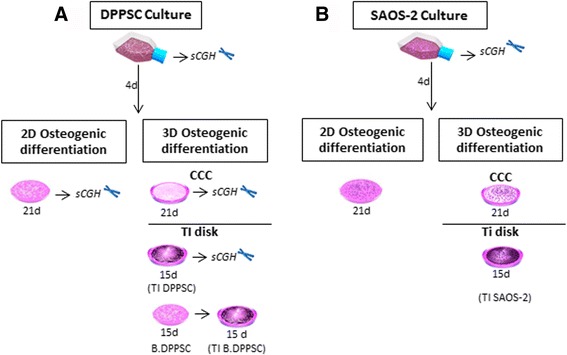
Schematic diagram of the experimental design. **a** Osteogenic differentiation of DPPSC cultures. Undifferentiated DPPSC were cultivated during 15 passages, from 6 different donors. The genetic stability was checked at passages 1, 5, 10 and 15 by sCGH. 2D osteogenic differentiation: DPPSC from 3 of the 6 donors at P10 were differentiated during 21 days with osteogenic media in plastic culture plates. 3D osteogenic differentiation: DPPSC from 2 of the same donors (at passages 1, 5 and 10) used also in 2D differentiation were differentiated on biomaterials (CCC and Ti disks) during 21 and 15 days. DPPSC after 15 days of osteogenic 2D differentiation (B.DPPSC) were also seeded on titanium disks and maintained during 15 days more in osteogenic medium (TI B.DPPSC). sCGH was performed in all DPPSC differentiations. **b** SAOS-2 at P10 were used as a control cell line in all experiments. Genetic instability of SAOS-2 was evaluated before osteogenic induction experiments. *DPPSC, Dental pulp pluripotent stem cells; SAOS-2, human osteosarcoma cell line; B.DPPSC, Bone-like DPPSC, differentiated 15 days in 2D conditions; TI DPPSC, DPPSC differentiated on titanium disks; TI B. DPPSC, Bone-like DPPSC differentiated on titanium disks; CCC, Collagen I-based Cell Carrier; TI disks, Ti6Al4V disks; sCGH, short-chromosome genetic hybridization*

In order to evaluate if DPPSC are appropriate to test the osteogenic capacity of well-known biomaterials, DPPSC from 2 of the same donors, used also in 2D differentiations, were differentiated on biomaterials. The chosen biomaterials, based on the extensive previous research using different types of cells, were collagen I based cell carriers (CCC) and titanium Ti6Al4V disks. The genetic stability was evaluated by sCGH following each differentiation step. A diagram of the experimental design is provided in Fig. 1.

#### Collagen CCC

The CCC sheets (Viscofan Bio Engineering) were equilibrated overnight in distilled water (200 μL per sheet) at 37 °C. The disks were transferred into 48-well plates preloaded with distilled water. After the removal of residual water, the culture plates containing the CCC sheets were dried overnight at RT under sterile conditions in a Laminar Air-Flow Cabinet. Before cell seeding, the dried CCC sheets were equilibrated with culture medium for 10 min at 37 °C. Due to the drying process, the collagen sheets firmly attached to the plastic well without the entrapment of air. Thus, cells could only adhere to the upper surface of the CCC. DPPSC were seeded at a density of 5 × 10^3^ cells/cm^2^ with osteogenic medium for 21 days. SAOS-2 cells were seeded and differentiated under the same conditions as a control.

#### Ti6Al4V disks

Ti6Al4V disks were obtained by cutting commercially available titanium alloy Ti6Al4V into 2.0-mm-thick disks with a 14 mm diameter and, subsequently, the surface was alumina-blasted and acid-etched to induce roughness, thus increasing the surface area (provided by MIS Implants Technologies Ltd.).

The osteogenic differentiation was performed on Ti6Al4V disks in 24-well plates at a density of 5 × 10^3^ cells/cm^2^ using osteogenic medium for 15 days.

For bone differentiation on titanium disks there are two models (see Fig 1). The first one, coded TI DPPSC, consist on undifferentiated DPPSC seeded directly on the disk surfaces. In the second model, coded as TI B.DPPSC, before being seeded on disks, DPPSC were differentiated for 15 days under 2D conditions, obtaining bone-like DPPSC (B.DPPSC). These osteogenic-like cells were then cultured on titanium disks and maintained during 15 days more in osteogenic medium.

### Immunohistochemistry

For DPPSC analysis, the cells were fixed with Cytofix (BD Biosciences) for 1 h at room temperature and then incubated with 1% Tween-20 for 30 min at room temperature to increase cell permeability. The slides were sequentially incubated overnight at 4 °C with primary antibodies against SSEA-4 (1:50, BD Pharmingen) or OCT3/4 (1:50, BD Pharmingen) and then for 60 min at 37 °C with PE-coupled anti-rabbit IgG and FITC-coupled anti-mouse IgG (1:200, BD Pharmingen). For differentiated DPPSC at day 21, primary antibodies against osteocalcin (1:50, Millipore) and collagen IV (1:100, Abcam) were used. Alexa 568 anti-rabbit IgG (1:200, Life Technologies) and Alexa 488 anti-mouse IgG (1:200, Santa Cruz Biotechnology) were used as secondary antibodies, respectively. Between each step, the slides were washed with Perm/Wash 1X (1:200, BD Biosciences). The cells were examined by confocal fluorescence microscopy.

### Flow cytometry

To confirm the phenotype of the undifferentiated DPPSC, FACS analysis was performed. The following fluorochrome labelled monoclonal antibodies were used: CD105-FITC (R&D Systems), CD29-PE (R&D Systems), CD146-FITC (BD Pharmingen), CD45-PE (BD Pharmingen), NANOG-FITC and OCT3/4-FITC (R&D Systems). To analyze the control samples, different IgG isotypes coupled to PE and FITC fluorochromes (BD Pharmingen) were used. The cells were suspended in PBS with 2% FBS and were incubated for 45 min at 4 °C in the absence of light. Subsequently, the cells were washed twice with 2% FBS-PBS and centrifuged for 6 min at 1800 rpm, thereby removing any residual fluorochrome to avoid false positive results. The pellets were re-suspended in volumes between 300 and 600 μl (depending on the number of cells) of PBS with 2% FBS. The flow cytometry measurements were made using a FACS cytometer (FACS Calibur, BD Biosciences) and analyzed with WinMDI 2.8 software. To detect and exclude nonspecific unions and auto fluorescence, at least 5x10^5^ cells were used for each sample.

### Western blot analysis

Total protein was extracted from undifferentiated DPPSC at passages 5, 10 and 15 using Trizol Reagent (Life Technologies) according to the manufacturer’s instructions. Protein quantification was performed using Bradford Reagent (Sigma). Aliquots of cell lysates at a concentration of 20 μg/μl were loaded on SDS-PAGE using 12% polyacrylamide gels and transferred onto nitrocellulose membranes. The membranes were then blocked with 1% (wt/vol) BSA in PBS containing 0.1% Tween-20. OCT3/4 and GAPDH primary antibodies (1:500, Abcam) were then incubated with the membranes, followed by washing and incubations with secondary antibodies (1:5000, Abcam). The primary antibodies used were anti-Osteocalcin (OC), anti-Osteopontin (OPN), anti-Collagen I (COL1) and anti-GAPDH as housekeeping (1:500, Abcam). The Western blot membrane was finally developed using Luminata Forte Western HRP substrate (Millipore).

### Short-comparative genomic hybridization (sCGH)

The sCGH technique was performed as described in Rius M. et al. [23] in triplicates of 12-14 cells from each sample. Undifferentiated DPPSC at passages 1, 5, 10 and 15 from 6 different donors were analyzed. Differentiated DPPSC in 2D conditions (well plates) were analyzed at passages 1, 5 and 10 from 3 patients after 21 days of osteogenic differentiation. Differentiated DPPSC, from 2 donors at passage 10, cultured on biomaterials (titanium disks and collagen carrier) were evaluated after 21 days of osteogenic differentiation.

SAOS-2 cells at passage 10 were analyzed before differentiation under 2D and 3D conditions. A 47, XXY sample was used to perform the hybridization of the controls and samples. This procedure was performed by an external service (Universitat Autònoma de Barcelona).

### Alkaline phosphatase (ALP) staining

ALP was determined in undifferentiated DPPSC (day 0) and after 3, 7, 15 and 21 days of DPPSC differentiation by ALP staining Kit (CosmoBio) following the manufacturer’s instructions. Briefly, cells were fixed with 10% Formalin Neutral Buffer Solution, for 20 min at RT. After 3 washes with 1 ml of deionized water, 200 μl of Chromogenic Substrate were added to each well and incubated for 20 min at 37 °C. Finally, to stop the reaction, the stainings were washed again with 1 ml of deionized water and observed the obtained blue staining under an optical microscope.

### Von Kossa Staining

Mineralization was determined in undifferentiated DPPSC (day 0) and after 3, 7, 15 and 21 days of DPPSC differentiation by Von Kossa Method for Calcium staining (Polysciences), following the manufacturer’s protocol. Briefly, cells were fixed with 10% Formalin Neutral Buffer Solution, for 20 min at RT and stained with 0.5 ml of 3% Silver Nitrate Solution under UV light for 45 min. After 3 washes with 0.5 ml of deionized water, 0.5 ml of 5% sodium thiosulfate were added to each well for 2 min at RT, and washed again with 1 ml of deionized water 3 times. Finally, the samples were counterstained in Nuclear Fast Red for 5 min at RT and photographed with an optic microscope.

### Alkaline phosphatase (ALP) activity

ALP activity was determined quantitatively in undifferentiated DPPSC (day 0) and after 7 and 15 days of differentiation in DPPSC, B. DPPSC and SAOS-2 cells on TI disks. An ALP Kit (BioSystems) was used in accordance with the manufacturer’s instructions. In short, the ALP was measured by determining the velocity of the formation of 4-nitrophenol starting from 4-nitrophenol phosphate. In order to initiate the ALP enzyme reaction, 20 μl of each sample supernatant were added to 1 ml of working solution containing 4:1 stock substrate solution (4-nitrophenol phosphate) and buffer solution (1.2 M diethanoloamine, 0.6 mM MgCl_2_). Finally, the absorbance of each sample was measured at 405 nm during 4 min. The increase of the absorbance was calculated with the average of absorbance per minute (ΔA/min).

### Scanning electron microscopy (SEM)

SEM analysis of the biomaterials was performed prior to differentiation and after DPPSC and SAOS-2 cells were differentiated on the biomaterials. The samples were fixed with 2.5% glutaraldehyde (Ted Pella Inc.) in 0.1 M Na-cacodylate buffer (EMS, Electron Microscopy Sciences, Hatfield, PA, USA) (pH 7.2) for 1 h on ice. After fixation, the samples were treated with 1% osmium tetroxide (OsO_4_) for 1 h. The samples were then dehydrated in serial solutions of acetone (30–100%) with the scaffolds mounted on aluminum stubs. The samples were examined with a Zeiss 940 DSM scanning electron microscope.

### RNA isolation, RT-PCR and qRT-PCR

Total RNA from undifferentiated cells, 2D differentiated cells (days 0, 7 and 21 days of differentiation) and differentiated cells on the biomaterials at the end of differentiation (day 21 in CCC and day 15 in Ti disks) was extracted by Trizol Reagent (Life Technologies) following the manufacturer’s instructions. RNA aliquots of 2 μg were treated with DNase I (Life Technologies) and reverse-transcribed using a Transcription First Strand cDNA Synthesis Kit (Roche), following the manufacturer’s instructions. PCR was performed using the primers listed in Table 1 for the amplification of OCT3/4, NANOG, SOX2, ALP, OC, COL1, RUNX2, VLA4, VCAM1, ITGα3, ITG and GAPDH cDNAs. The resulting amplicons were resolved by agarose gel electrophoresis. A human bone cDNA sample (Biocompare) and SAOS-2 cells were used as a positive control.

**Table 1:** Primer pairs used to assess osteoblast differentiation in the RT-PCR and qRT-PCR amplification

Gene	Accession number	Forward primer (5′-3′)	Reverse primer (5′-3′)	Product size (bp)	Use
OCT 3/4	NM_002701	GTGGAGAGCAACTCCGAT G	TGCAGAGCTTTGATGTCCTG	122	RT-PCR qRT-PCR
NANOG	NM_024865	CAGAAGGCCTCAGCACCTAC	ATTGTTCCAGGTCTGGTTGC	111	RT-PCR qRT-PCR
ALP	NM_000478	GGACATGCAGTACGAGCTGA	GTCAATTCTGCCTCCTTCCA	133	RT-PCR
ALP	NM_000478	CCGTGGCAACTCTATCTTTGG	GCCATACAGGATGGCAGTGA	79	qRT-PCR
COL1	NM_000088	ACTGGTGAGACCTGCGTGTA	CAGTCTGCTGGTCCATGTA	263	RT-PCR
COL1	NM_000088	CCCTGGAAAGAATGGAGATGAT	ACTGAAACC TCTGTGTCCCTTCA	139	qRT-PCR
OC	NM_199173	GTGCAGCCTTTGTGTCCAA	GCTCACACACCTCCCTCCT	129	RT-PCR
OC	NM_199173	AAGAGACCCAGGCGCTACC T	AAC TCGTCACAGTCCGGATTG	110	qRT-PCR
RUNX2	NM_001146038	TTACTGTCATGGCGGGTAAC	GGTTCCCGAGGTCCATCTA	220	RT-PCR
RUNX2	NM_001146038	AGCAAGGTTCAACGATCTGAGAT	TTTGTGAAGACGGTTATGGTCAA	81	qRT-PCR
VLA4	NM_000885	CGAACCGATGGCTCCTAGTG	CACGTCTGGCCGGGATT	115	RT-PCR
ITGα3	NM_002204	TCCGAGTCAATGTCCACAGA	GCTGGGCTACCCTATTCCTC	88	RT-PCR qRT-PCR
ITGαV	NM_002210	CCTTGCTGCTCTTGGAAC TC	ATTCTGTGGCTGTCGGAGAT	74	RT-PCR qRT-PCR
GAPDH	NM_002046	CTGGTAAAGTGGATATTGTTGCCAT	TGGAATCATATTGGAACATGTAAACC	81	RT-PCR qRT-PCR

*OCT-3/4* octamer-binding transcription factor 4, *Nanog* Nanog homeobox, *ALP* alkaline phosphatase, *COL1* type I collagen, *OC* osteocalcin, *RUNX2* Runt-related protein 2, *VLA-4* Integrin alpha4beta1, *ITGα3* integrin alpha 3, *ITGαV* integrin, alpha V, *GAPDH* glyceraldehyde-3-phosphate dehydrogenase

Quantitative RT-PCR was performed using a CFX96 thermocycler (Bio-Rad). A total of 50 ng of cDNA of TI DPPSC, TI B.DPPSC and TI SAOS-2 cells on Ti disks was used. cDNA samples were amplified using specific primers and SYBR Green Supermix (Bio-Rad Laboratories, Inc.). The expression levels of the genes of interest (OCT3/4, RUNX2, COL1, OC, VLA4, ITGα3 and ITGαV) were normalized against the housekeeping gene GAPDH. The relative expression levels were normalized to day 0 of the DPPSC cDNAs in 2D differentiation or with TI SAOS-2 cDNAs in titanium differentiation, which were assigned as 1. All analyses were performed using the 2^-ΔΔCT^ method and 3 technical replicates.

### Statistical analysis

Data from the osteogenic differentiation (qRT-PCR, calcium quantification, ALP activity) of DPPSC on 2D and titanium were analyzed by applying two-way analysis of variance or ANOVA for multiple factors. Statistical analysis was performed using the SPSS 21.0 software package. Confidence intervals were fixed at 95% (*P < 0.05*).

## Results

### Characterization of dental pulp pluripotent-like stem cells

DPPSC were isolated as a new subpopulation of DPSC with pluripotent-like characteristics. They were expanded and characterized as previously described [22]. The results with their pluripotent-like characteristics during 15 culture passages comparing with DPMSC are shown in Additional file 1: Figure S1.

### 2D Osteogenic differentiation

**Fig. 2:**
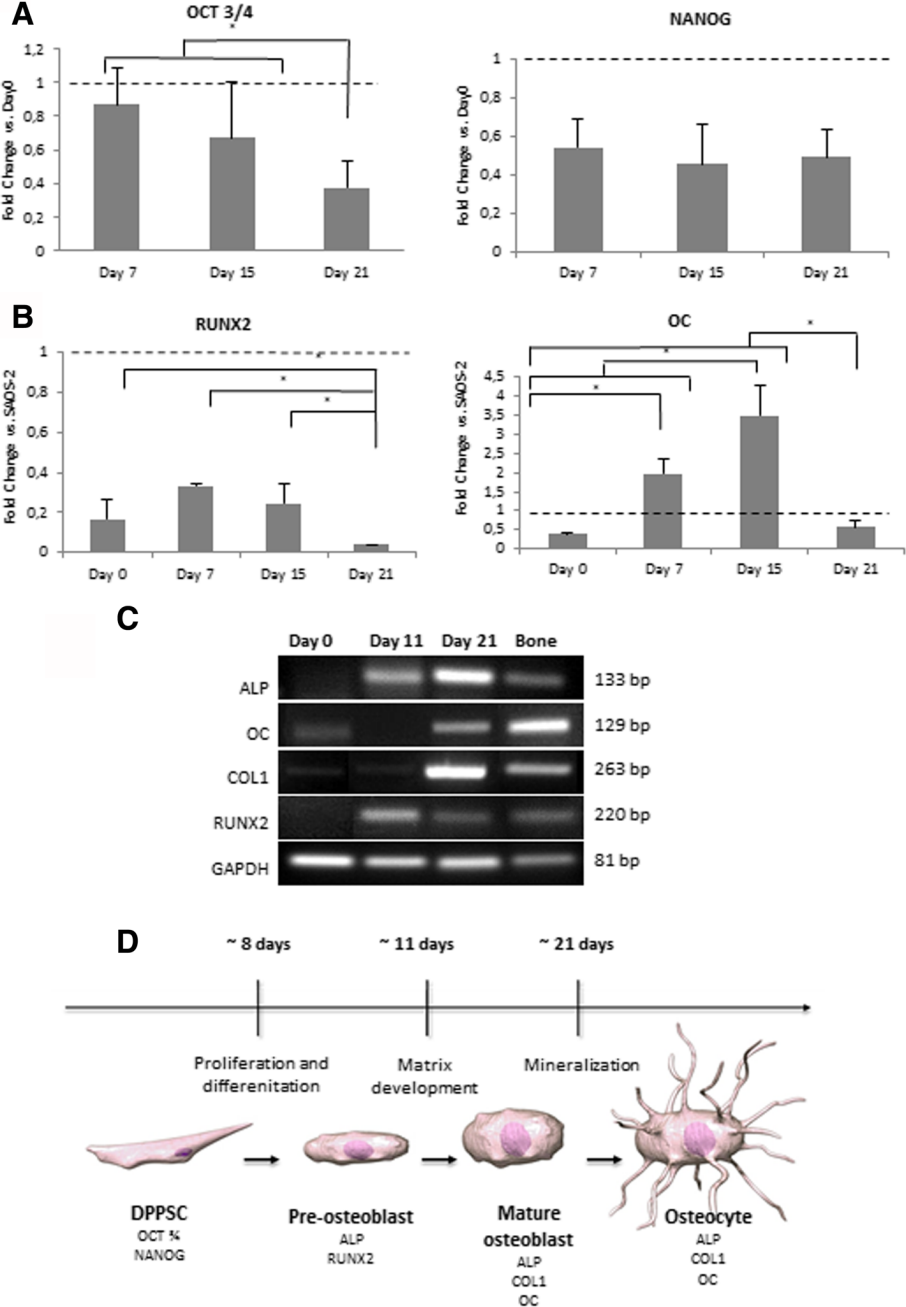
Stages of DPPSC osteogenic differentiation during 21 days. **a** Relative expression of pluripotency (OCT3/4, NANOG) markers during the osteogenic differentiation of DPPSC versus DPPSC at Day 0 (dotted line). **b** Relative expression of osteogenic markers (RUNX2, OC) during the osteogenic differentiation of DPPSC versus SAOS-2 cells differentiated over 21 days in osteogenic medium (*dotted* line). **c** RT-PCR for osteogenic markers (ALP, OC, COL1, RUNX2) at days 0, 11 and 21 of differentiation. Bone cDNA was used as a positive control and GAPDH was used as a housekeeping. **d** Proposed scheme for osteogenic DPPSC culture with recognizable stages of differentiation. *P < 0.05

Under 2D conditions, the osteogenic differentiation of DPPSC was investigated by examining the gene expression profile of pluripotent and osteogenic markers during 21 days of culture in osteogenic medium (Fig. 2a-b). Firstly, qRT-PCR analysis revealed a gradual decrease in the expression of the pluripotency markers OCT3/4 and NANOG during DPPSC differentiation (Fig. 2a). On the other hand, the transcript expression of the early osteogenic marker RUNX2 was shown to increase and peaked at day 7, showing notably lower expression at the end of the differentiation. Then, the OC expression was up-regulated reaching the highest levels at day 15 of differentiation (Fig. 2b).

Moreover, RT-PCR results showed that the expression levels of ALP and COL1 were pronouncedly increasing until week 3 and confirmed that RUNX2 expression was decreasing after first week of differentiation (Fig. 2c). Finally, in order to summarize the genetic pattern during the osteogenic differentiation of DPPSC, a schematic representation with the different stages was performed (Fig. 2d).

**Fig. 3:**
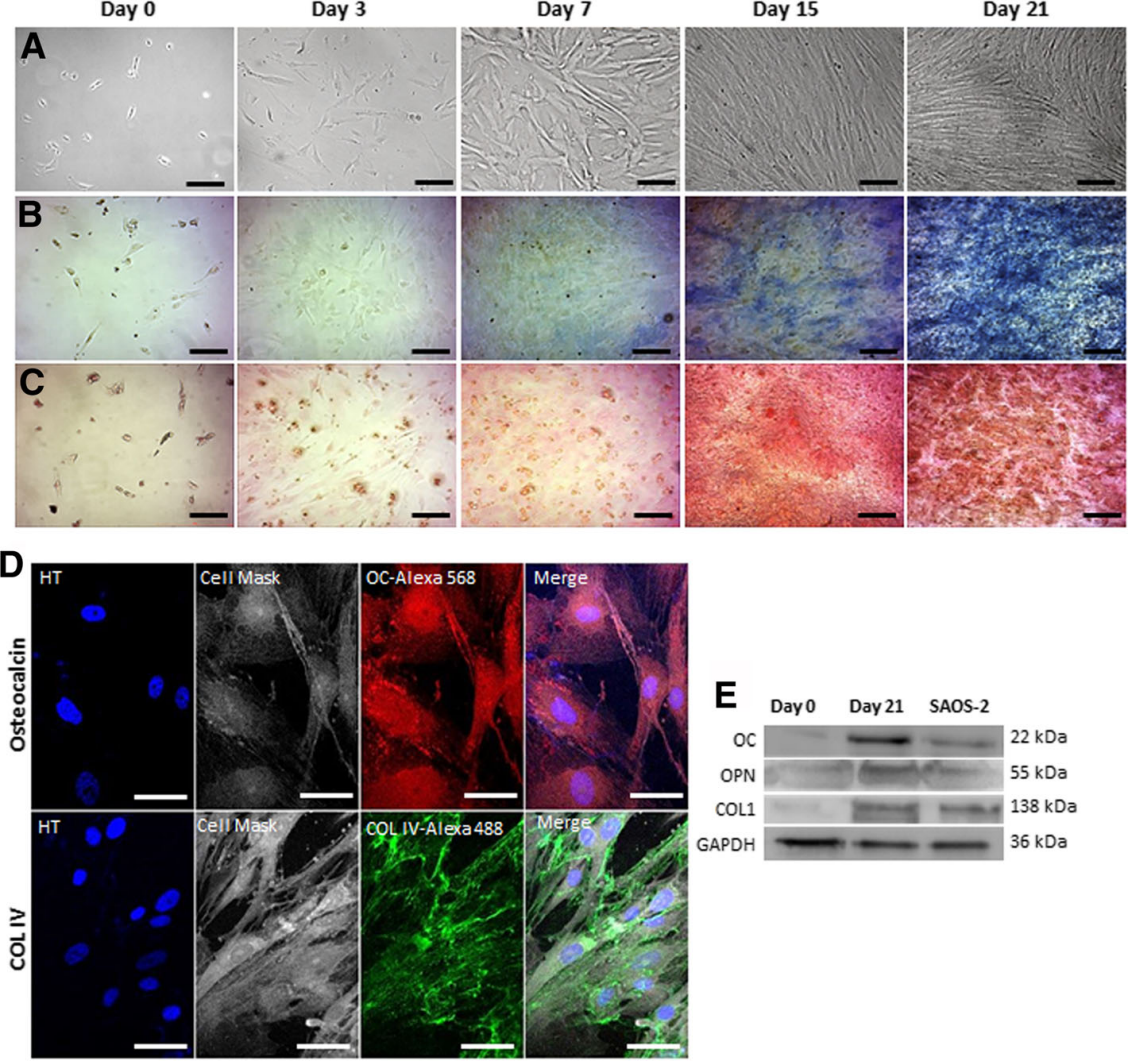
Osteogenic capacity of DPPSC during 21 days. **a** Cell morphology of DPPSC observed with optic microscopy at days 0, 3, 7, 15 and 21 of osteogenic differentiation. **b** ALP activity observed with optic microscopy at days 0, 3, 7, 15 and 21 by an ALP staining (*blue*). **c** Images of mineralitzation at days 0, 3, 7, 15 and 21 stained by the von Kossa method showing mineralized bone (*brown*) and osteoid, supporting tissue and structures (*red*). **d**, **e** Osteogenic protein expression of DPPSC after 21 days. **d** Immunofluorescence analysis of DPPSC showing the expression of Osteogenic markers at day 21 of differentiation. Hoechst (HT) as a nucleus control. Scale bars: 200 μm (**a**-**c**), 50 μm (**d**). **e** Western Blot analysis of osteogenic markers at day 0 and 21 of differentiation. SAOS-2 cells were used as a positive control. GAPDH was used as a housekeeping control

In addition, DPPSC morphology was examined by optical microscopy along the osteogenic differentiation at different time points. At day 0, undifferentiated DPPSC were small-sized cells with rounded morphology, large nuclei and low cytoplasm content. By the second week of differentiation DPPSC adopted an elongated morphology and started to mineralize (Fig. 3a).

Furthermore, functional osteogenic activity was qualitatively detected at different time points. ALP activity enhancement was clearly observed in a time dependent manner over the differentiation process (Fig. 3b). Moreover, as a mineralization assay, von Kossa staining showed the development of red regions which were rich in osteoid as well as, detected in brown, calcium phosphate depositions (Fig. 3c).

In order to corroborate the qRT-PCR results with the levels of protein expressed and hence, to evaluate the differentiation efficiency of DPPSC, the protein expression of the osteogenic markers was analyzed by immunofluorescence and Western blot analyses. Immunofluorescence analysis using specific anti- OC and Col IV antibodies showed the expression of the OC localized in the cytoplasm and the formation of the extracellular collagen matrix (Fig. 3d). In addition, higher protein expression of OC, OPN and COL 1 was detected at day 21 comparable to that observed in SAOS-2 cells (Fig. 3e).

### Genetic stability

**Fig. 4:**
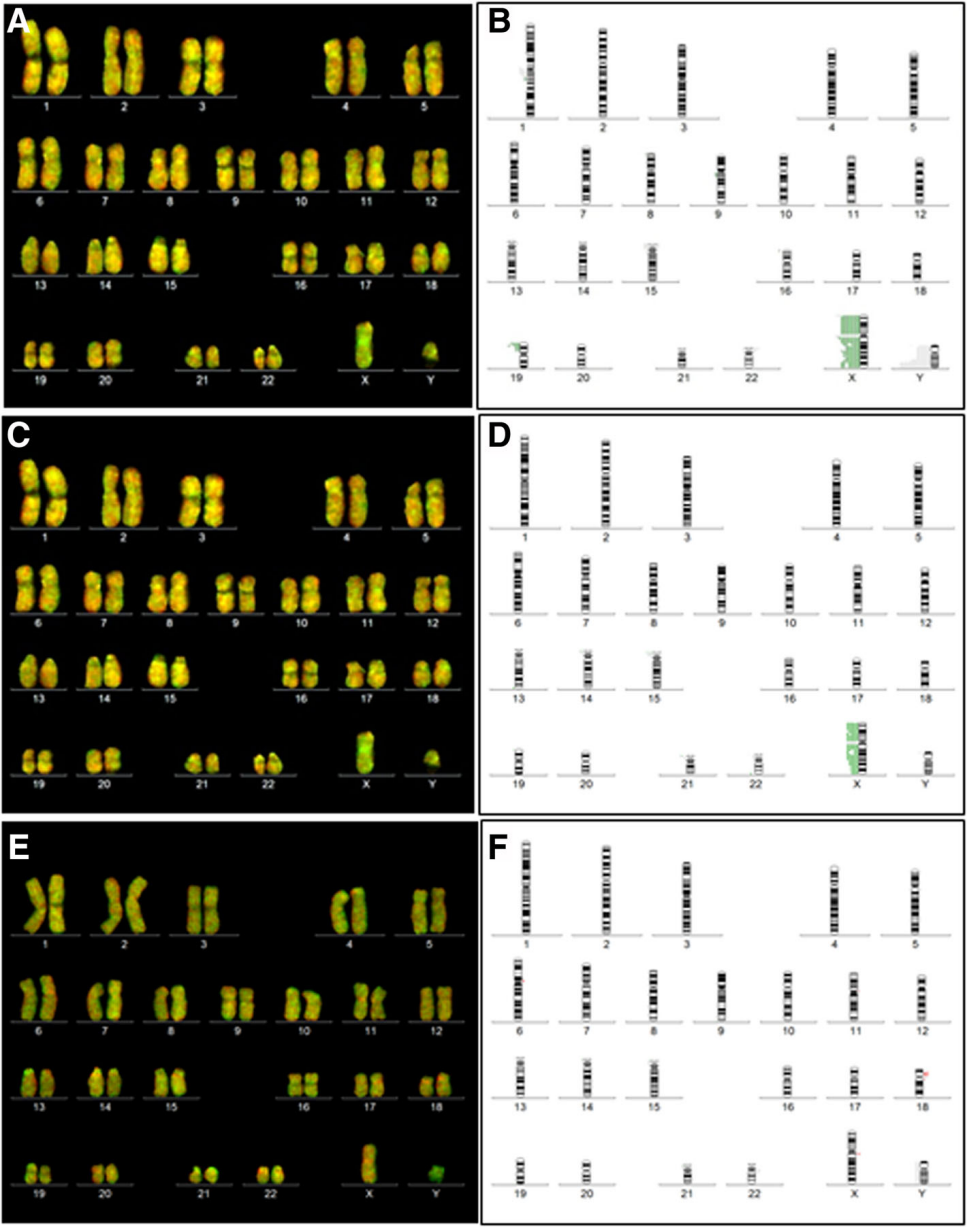
Genetic stability of DPPSC, before and after osteogenic differentiation by sCGH analysis. **a**, **b** sCGH from undifferentiated DPPSC at passages 1, 5, 10 and 15 (*N* = 6, XX and XY donors); **a** Fluorochromes image; **b** Fixed limits summary. **c**, **d** sCGH at day 21 of 2D osteogenic differentiation from DPPSC at passages 1, 5 and 10 (*N* = 3, XX and XY donors). **c** Fluorochromes image; **d** Fixed limits summary. **e**, **f** CGH from DPPSC (P10) at day 21 of osteogenic differentiation on biomaterials: Collagen and Titanium carriers (*N* = 2, XX donors). **e** Fluorochromes image; **f** Fixed limits summary. 47, XXY control samples (labeled in *green*) and DPPSC samples (labeled in *red*) were mixed and co-hybridized onto 12 (46, XY) metaphases in triplicate. A gain in the X or Y chromosome dosage was due to sex differences

The genetic stability was checked in undifferentiated DPPSC and during their differentiation into bone-like tissue on culture plates and on biomaterials by sCGH. Undifferentiated and differentiated DPPSC exhibited a normal karyotype with no presence of any aneuploidy or any chromosome structural alteration. Therefore, results showed that DPPSC maintained the stability before and after the differentiation process (Fig. 4a-d).

Moreover, sCGH confirmed a normal karyotype of DPPSC cultured on CCC and Ti6Al4V disks after differentiation (Fig. 4e-f). In contrast, SAOS-2 cells showed genetic mutations in numerous chromosomes, probably due to their carcinogenic origin (Additional file 2: Figure S2).

### Osteogenic differentiation on Biomaterials

#### Osteogenic differentiation on Collagen I-based Cell Carrier (CCC)

**Fig. 5:**
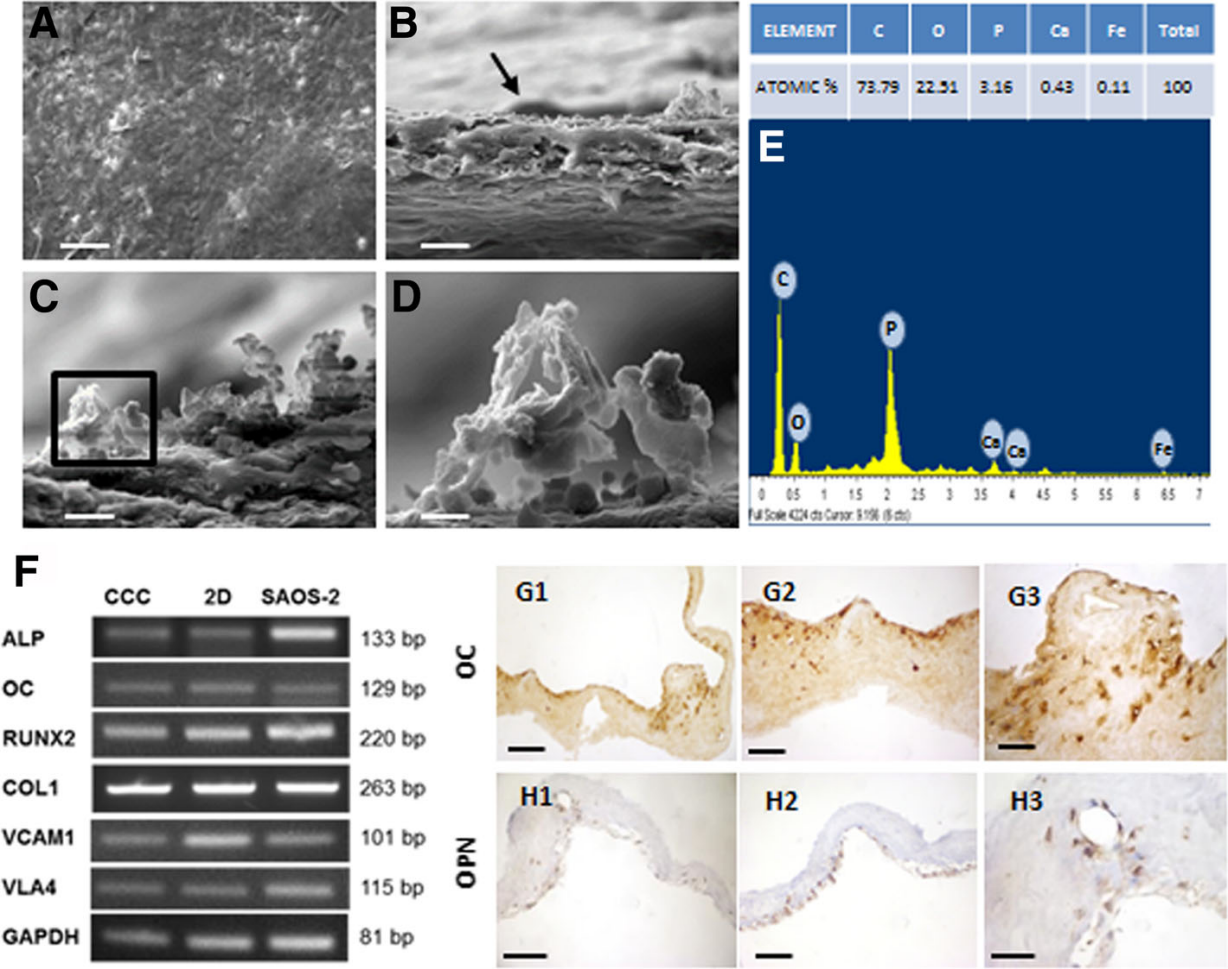
Osteogenic differentiation on Collagen I-based Cell Carrier (CCC). **a**, **b** SEM images of differentiated DPPSC adhered on CCC surface (*black* arrow). **c** SEM image of differentiated DPPSC with hydroxyapatite deposition on CCC surface. **d** SEM image of hydroxyapatite deposition on CCC. Scale bars: 100 μm (**a**), 20 μm (**b**), 10 μm (**c**), 2 μm (**d**). **e** Microanalysis of the CCC surface with atomic concentrations. **f** RT-PCR gene expression analysis of differentiation markers (OC, ALP, COL1) and adhesion markers (VCAM1, VLA4) in DPPSC cultured on CCC, DPPSC cultured on 2D (plastic surface) and SAOS-2 cultured on CCC. GAPDH was used as a housekeeping. **g**, **h** Immunohistochemistry of differentiation markers (OC, OPN) in differentiated DPPSC on CCC. Scale bars: 1000 μm (**g1**, **h1**), 400 μm (**g2**, **h2**); 200 μm (**g3**, **h3**)

DPPSC were cultured on CCC and the differentiation was induced for 21 days (Fig. 5). SEM was utilized to visualize the scaffold/cells constructs and obtain a better understanding of the cell morphology. CCC scaffolds with the attached cells exhibited a dense microstructure. The cells were well dispersed and attached, covering the entire surface and penetrating inside the collagen scaffold (Fig. 5a-b).

Moreover, SEM images showed an extra cellular matrix formed by calcium phosphate depositions (Fig. 5c-d), also confirmed by an atomic microanalysis; with the presence of calcium and phosphorus ions, 0,43% and 3.16%, respectively (Fig. 5e). In addition, RT-PCR analysis for RNA extracted after the osteogenic induction, revealed an enhancement of the osteogenic markers ALP, OC and RUNX2 as well as adhesion markers, COL1, VCAM1 and VLA4 (Fig. 5f) at similar levels to that of 2D- differentiated DPPSC or SAOS-2 cells. In addition, immunohistochemistry sections using specific OC and OPN antibodies showed the presence of these important proteins implied in the mineralization process (Fig. 5g-h).

#### Osteogenic differentiation on titanium alloy disks (TI disks)

DPPSC (TI DPPSC), 15 day bone-like DPPSC (TI B.DPPSC) and SAOS-2 cells (TI SAOS-2) were cultivated and differentiated on titanium alloy disks with osteogenic medium for 15 days.

**Fig. 6:**
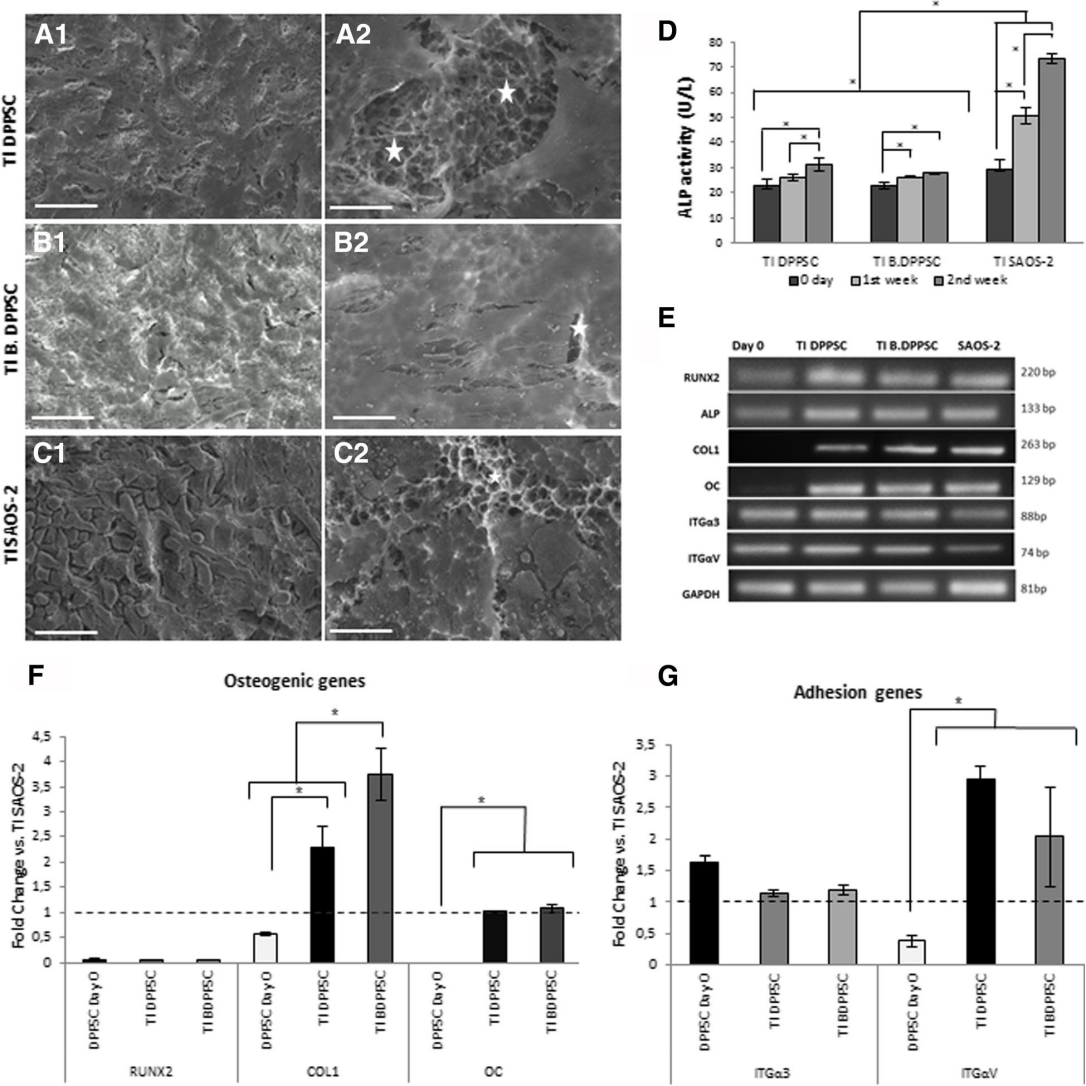
Osteogenic differentiation on Ti disks. **a-c** SEM images of the different cells types differentiating on Ti alloy disks. (**a**) DPPSC, (**b**) Bone-like DPPSC, (**c**) SAOS-2. Stars indicate the Ti surface without cells. Scale Bars: 40 μm (**a1**, **b1**, **c1**), 10 μm (**a2**, **b2**, **c2**). **d** ALP activity (U/L) of TI DPPSC, TI B.DPPSC and TI SAOS-2 differentiating on Ti alloy disks at week 1 and 2 (*n* = 3). **e**-**g** RT-PCR (**e**) and qRT-PCR (**f**, **g**) gene expression analysis of differentiation (RUNX2, COL1 and OC) and adhesion markers (ITGα3 and ITGαV) at second week of cell differentiations on Ti alloy disks (*n* = 3). TI SAOS-2 was used for normalization. DPPSC at day 0 of differentiation were used as a negative control and GAPDH was used as housekeeping gene. *P < 0.05

SEM micrographs showed a high-density cell mass on the surface of the disk for all cell populations, indicating that the cells adhered and grew favorably (Fig. 6a-c). Moreover, TI B.DPPSC seemed to cover more surface than the other cell types (Fig. 6b2). The results of the ALP assay showed that the ALP activity increased significantly over time in TI DPPSC, TI B.DPPSC and TI SAOS-2, demonstrating that the cells acquired this functional activity during osteoblast differentiation (Fig. 6d). The behavior of TI DPPSC and TI B.DPPSC was similar; differences between cell types were only statistically significant when comparing TI SAOS-2 cells with the other cell types.

To further characterize the differentiation status of the cells, the expression levels of several osteogenic and adhesion markers were evaluated at the end of the osteogenic differentiation process on TI disks using SAOS-2 cells for normalization (Fig. 6e-g). Results showed that TI DPPSC and TI B.DPPSC showed less expression of the early osteogenic marker RUNX2 than SAOS-2 and more expression of the osteogenic advanced markers COL1 and OC, with the highest expression in TI B.DPPSC (Fig. 6f). Furthermore, the analyses of the adhesion markers showed higher expression of the integrin genes in DPPSC than in SAOS-2 cells (Fig. 6g), confirming the high adhesion potential of DPPSC to the titanium surface.

## Discussion

The third molar represents a very accessible organ, which is often extracted for dental reasons, and due to its late development, allows the presence of progenitor cells. Previously, we identified DPPSC as a new subpopulation of DPSC cultivated in a media containing LIF, EGF and PDGF to maintain their pluripotent state. In addition we showed that DPPSC are able to differentiate into adult tissues generating all three embryonic germ layers, i.e., endothelial cells, neurons, bone and hepatocyte-like cells [9].

DPMSC and DPPSC are obtained from the same dental pulp but cultured at different cell densities and different medium conditions. In this study, the phenotypical analysis showed high expression levels of CD29 and CD105 markers, and low expression levels of CD45, indicating that DPPSC share several similarities with DPMSC. Nevertheless, DPPSC also express pluripotency markers such as OCT3/4, NANOG, SOX2 and SSEA4, which are indispensable for infinite stem cell division without affecting differentiation potential. On the other hand, we consider that pluripotent stem cells have higher value when testing the differentiation capacity of biomaterials, since these cells provide highly undifferentiated cells which need to be guided merely with the biomaterial. Hence, we consider that the use of osteoblastic-like cells to determine the osteogenic capacity of biomaterials is meaningless compared to the use of pluripotent-like cells. For all these reasons, we elected DPPSC to perform the osteogenic differentiations on biomaterials.

To address the requirement to well-characterize the DPPSC differentiation process, one of the first aims of this study was to analyze the model system for osteogenesis of this new stem cell population and to establish the expression profile of bone related genes during their differentiation process.

In general, our results indicate that the osteogenic differentiation of DPPSC is in accordance with the human osteoblastic development, which can be divided into three chronologically stages: proliferation, matrix development and mineralization [24, 25]. Thus, RUNX2 is expressed during early osteoblast differentiation and is strictly required for the differentiation and appropriate functioning of osteoblasts [26]. Here, we found that RUNX2 was highly expressed during the first week of DPPSC differentiation, while the expression levels of pluripotency markers OCT3/4 and NANOG were reduced, indicating the end of the proliferation stage. At the second week, during the extracellular matrix maturation, RUNX2 expression levels were progressively down-regulated while differentiated cells began to express OC, in agreement with the reports that RUNX2 is an upstream gene of OC [25, 26]. Moreover, there was a remarkable peak of OC at day 15 that indicated an early mineralization of the differentiated DPPSC. On the other hand, by comparing the gene expression of differentiated DPPSC with differentiated SAOS-2, we can observe that SAOS-2 showed more expression of the initial osteogenic marker RUNX2 and similar levels of the advanced marker OC. It was probably due to the uncontrolled proliferation rate of SAOS-2, which constantly produces immature cells. SAOS-2 cells have been frequently used in applied biology since they are from human origin, they provide an unlimited number of cells and therefore, they are a fast and a cheap cell model to test osteogenesis in biomaterials. However, we consider that the use of pluripotent-like cells that can potentially differentiate into any lineage assesses better the role of the biomaterial in the osteogenic differentiation. Moreover, as DPPSC are in a previous stage of differentiation than SAOS-2 cells, this allows the analysis of the osteogenic differentiation since the beginning of the process.

Furthermore, RT-PCR analysis revealed that ALP was also expressed during the phase of matrix development, increasing from the first week until the end of the differentiation. This triggers the mineralization stage, commonly observed by the production of hydroxyapatite crystals [27].

At the same time, COLI was detected during the matrix development until the mineralization stage indicating that the maturation process of osteoblast-like cells produced abundant matrix proteins that were deposited as osteoid or non-mineralized bone matrix [28]. In addition, results showed very similar osteogenic expression levels between differentiated DPPSC and human bone cDNA, in accordance with a previous study of the osteogenic differentiation of DPPSC, where the bone-like tissue formed by DPPSCs in 3D were similar in complexity to human bone tissue [8].

In order to assess the functional activity of bone-like DPPSC, mineralization and ALP stainings were performed during DPPSC differentiation. By the third week, both stainings demonstrated an osteoid mineralization by the accumulation of calcium phosphate in the form of hydroxyapatite. In addition, protein analysis with high expression of late osteogenic markers, OC and OPN, confirmed the osteocyte-like phenotype of DPPSC. In summary, our results indicated that differentiated DPPSC show a behavior pattern similar to human primary osteoblasts [2, 29]. These results support the capacity of DPPSC to differentiate into bone-like tissue similar to the traditionally used osteoblastic progenitors.

After analyzing the expression profile of osteogenic genes during DPPSC differentiation, we examined the genetic stability of this pluripotent-like stem cell population before and after their differentiation process on biomaterials. The rationale was based on previous studies that used adult stem cells with genetic stability to assess the quality and osteogenic capacity of biomaterials [8, 30]. Thus, we analyzed the genetic stability of DPPSC and SAOS-2 by sCGH, a direct aneuploidy screening that allows the detection of chromosome imbalances generated by aberrant segregation and structural differences for fragments larger than 10–20 Mb [31]. Our results showed a normal chromosomal dosage during DPPSC culture expansion until passage 15. This was also evident at the end of the differentiation process, both in culture plates and on biomaterials. Nevertheless, we confirmed some genetic instability in SAOS-2 cells that have been probably related to the progression and genesis of osteosarcoma. This low stability of SAOS-2 could induce phenotype alterations, aberrations in mitotic processes or lack of growth inhibition affecting the results of biomaterial testing [5]. Furthermore, some reports demonstrate a direct correlation between culture density and the occurrence of DNA damage and genomic alterations during the culture of stem cells in vitro [32]. These effects are largely caused by the accumulation of lactic acid in the culture medium and the associated medium acidification [32, 33]. Here, the particular culture conditions of DPPSC (medium composition, low serum levels, low cell confluence before passaging and low cell culture density) could facilitate the preservation of the genomic stability, making DPPSC a more stable cell model for testing biomaterials in bone regeneration studies.

It is known that natural materials, metals and synthetic polymers scaffolds organize stem cells into complex spatial groupings which mimics native tissue [34]. In this study, we evaluated the capacity of DPPSC to grow and differentiate in a natural collagen scaffold (CCC). Natural biological and mechanical properties of native collagen provides a bio-mimetic environment for stem cells as well as a mechanical support, providing collagen based biomaterials as commonly used support for cell culture and tissue engineering [35]. Analysis of the scaffold surface and cell morphology by electron microscopy emphasized the high affinity of DPPSC to grow in CCC, the homogenous cell distribution and the high level of calcification at the final stage of the differentiation. The expression of osteogenic and adhesion markers in differentiated DPPSC in CCC was comparable to DPPSC differentiated on culture plates or SAOS-2 cultured in CCC. Moreover, an immunohistochemistry assay showed the expression of advanced osteogenic markers (OC, OPN), corroborating the presence of osteocyte-like cells and the complete differentiation and biocompatibility of DPPSC over CCC in vitro.

On the other hand, it has been demonstrated that metal scaffolds are also suitable for hard-tissue applications. The loosening of implants from bone tissues has been a problem in reconstructive surgery and joint replacement. Brånemark introduced the term “osseointegration” to describe this modality for stable fixation of titanium to bone tissue [36]. Some studies report that titanium disks favor the osseointegration, stimulating the functions of osteoblasts on the surface, such as the adhesion, proliferation or secretion of specific proteins composing the matrix [37]. The most common cell sources used for this propose, are immortalized cell lines or fibroblasts [38, 39]. However, these cell types show different characteristics from stem cells involved in bone regeneration in vivo*.* Currently, there are few studies regarding the osseointegration of titanium with stem cells [40]. Therefore, this study attempts to evaluate the osteogenic capacity of TI disks with an adult pluripotent-like stem cell population. For this purpose, two populations of DPPSC were used: DPPSC and B.DPPSC. B.DPPSC were DPPSC pre-differentiated for 15 days, when they reached the beginning of the mineralization stage, corresponding with the peak of OC expression. This previous differentiation of the cells on plates before transferring to titanium surfaces can improve the evaluation of biomaterials, reducing the costs (differentiated cells expand more rapidly and can be cultured at higher densities) and accelerating the process.

SEM images obtained of TI disks showed a complete coverage of the surface with all cell types, suggesting a major density, expansion and adhesion of TI B.DPPSC. Furthermore, at the second week of differentiation, ALP concentration increased in all cell types, indicating a mineralization stage with the development of a calcified matrix at 15 days of osteogenic induction. On the other hand, the high ALP activity levels in SAOS-2 could be explained by some studies which revealed that this property of SAOS-2 can differ considerably from primary osteoblasts behavior [2].

At the end of differentiation, TI SAOS-2 cells showed higher expression of initial markers ALP and RUNX2 and lower expression of advanced markers COL1 or OC. These results could indicate that this carcinogenic cell line presents cells in a more immature stage than DPPSC lines, probably due to their constant and uncontrolled proliferation rate. Moreover, we found that the expression of COL1, the most important protein in the non-mineralized matrix, was higher in TI B.DPPSC. Finally, TI DPPSC and TI B.DPPSC demonstrated also a higher expression of the adhesion markers than TI SAOS-2 cells, suggesting that DPPSC had a strong capacity to adhere on titanium surfaces.

## Conclusions

In conclusion, these results support the use of DPPSC, a new pluripotent-like subpopulation of DPSC, as a good alternative model to evaluate the biocompatibility and the differentiation capacity of different types of biomaterials commonly used for bone regeneration studies. DPPSC showed high osteogenic and adhesion potential whilst seemed to maintain genetic stability during culture expansion and differentiation. However, further studies assessing their genetic stability by means of more accurate methods and their in vivo biocompatibility will be necessary before testing them in clinical applications.

## Additional files

Additional file 1: Figure S1. Characterization of undifferentiated DPPSC. **a** Cell morphology of DPPSC from passage 10 observed with optic microscopy. DPPSC are characterized as small-sized cells with large nuclei and low cytoplasm content. **b** Immunofluorescence analysis of OCT3/4-FITC, SSEA4-PE, and Merge. Hoechst (HT) as a nucleus control. DPPSC were positive for these embryonic markers, and both were located in the nucleus. **c** FACS analysis of DPPSC.** c1** FACS analysis of membrane markers: CD105 (92,15%), CD29 (99,63%), CD146 (15,54%) and CD45 (0.04%). **c2** FACS analysis of pluripotency nuclear markers: OCT3/4 (76,72%) and NANOG (30,18%). **d** RT-PCR of OCT3/4, NANOG and SOX2 expresions in DPPSC and DPMSC. **e** Western Blot analysis of OCT3/4 in DPPSC and DPMSC at different time points (5, 10 and 15 passages). GAPDH as a housekeeping. (TIF 1031 kb)

Additional file 2: Figure S2. sCGH summary from SAOS-2 cells at passage 10. 47, XXY control samples (labelled in green) and SAOS-2 samples (labelled in red) were co-hybridized onto 46, XY metaphases. (TIF 234 kb)

## Abbreviations

ALP: Alkaline phosphatase
B.DPPSC: Bone-like DPPSC 15 days differentiated
CCC: Collagen I-based Cell Carrier
DPMSC: Dental pulp mesenchymal stem cells
DPPSC: Dental pulp pluripotent-like stem cells
DPSC: Dental pulp stem cells
SAOS-2: Human osteosarcoma cell line
sCGH: Short-comparative genomic hybridization
TI B.DPPSC: B.DPPSC differentiating on TI alloy disks
TI DPPSC: DPPSC differentiating on Ti alloy disks
TI SAOS-2: SAOS-2 differentiating on Ti alloy disks
