# Supplementary figures and images for: Dental pulp pluripotent-like stem cells (DPPSC), a new stem cell population with chromosomal stability and osteogenic capacity for biomaterials evaluation
Source: BMC Cell Biol. 2017 Apr 21;18:21. doi: 10.1186/s12860-017-0137-9 (PMC5399345; doi:10.1186/s12860-017-0137-9)

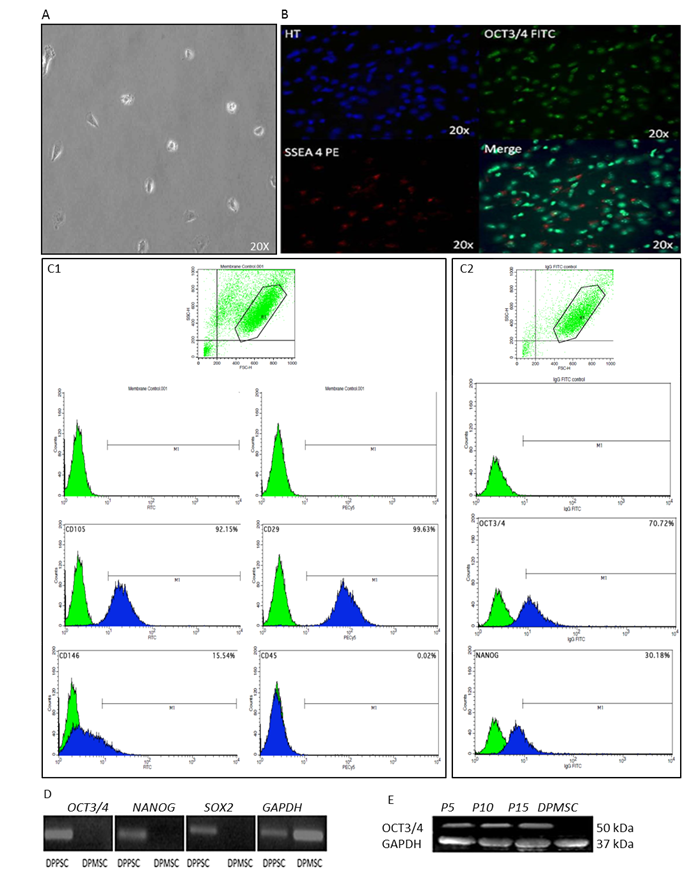

Supplement: Supplementary file 1 — Characterization of undifferentiated DPPSC. a Cell morphology of DPPSC from passage 10 observed with optic microscopy. DPPSC are characterized as small-sized cells with large nuclei and low cytoplasm content. b Immunofluorescence analysis of OCT3/4-FITC, SSEA4-PE, and Merge. Hoechst (HT) as a nucleus control. DPPSC were positive for these embryonic markers, and both were located in the nucleus. c FACS analysis of DPPSC. c1 FACS analysis of membrane markers: CD105 (92,15%), CD29 (99,63%), CD146 (15,54%) and CD45 (0.04%). c2 FACS analysis of pluripotency nuclear markers: OCT3/4 (76,72%) and NANOG (30,18%). d RT-PCR of OCT3/4, NANOG and SOX2 expresions in DPPSC and DPMSC. e Western Blot analysis of OCT3/4 in DPPSC and DPMSC at different time points (5, 10 and 15 passages). GAPDH as a housekeeping. (TIF 1031 kb) [file 12860_2017_137_MOESM1_ESM.tif]

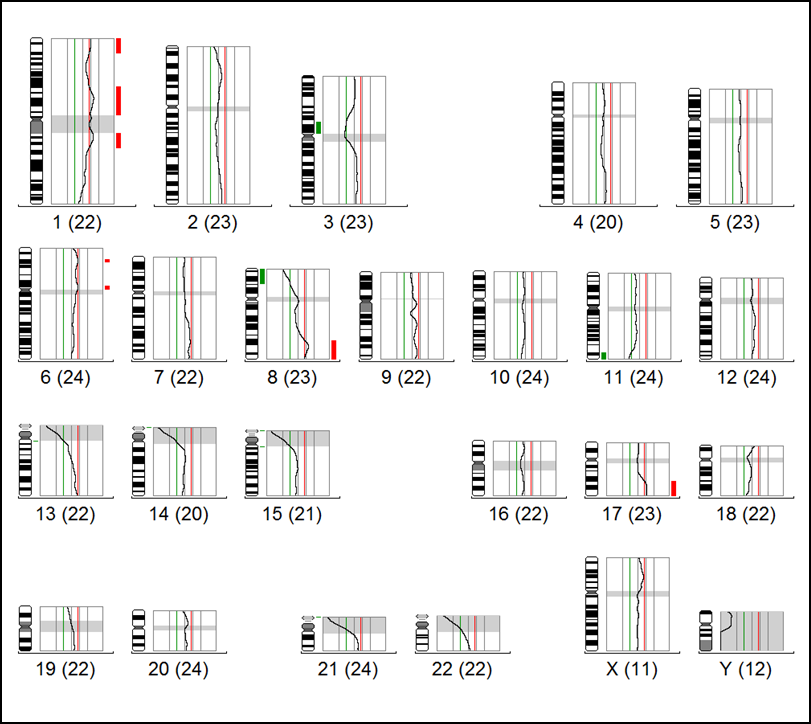

Supplement: Supplementary file 2 — sCGH summary from SAOS-2 cells at passage 10. 47, XXY control samples (labelled in green) and SAOS-2 samples (labelled in red) were co-hybridized onto 46, XY metaphases. (TIF 234 kb) [file 12860_2017_137_MOESM2_ESM.tif]
